# Case Report: Surgical management of primary lymphedema with a novel PROX1 mutation involving upper and lower limbs

**DOI:** 10.3389/fgene.2025.1560471

**Published:** 2025-07-03

**Authors:** Junzhe Chen, Liangliang Wang, Xiangkui Wu, Bihua Wu, Hai Li, Shune Xiao, Chengliang Deng

**Affiliations:** ^1^ Department of Burns and Plastic Surgery, Affiliated Hospital of Zunyi Medical University, Zunyi, Guizhou, China; ^2^ The Collaborative Innovation Center of Tissue Damage Repair and Regeneration Medicine, Zunyi Medical University, Zunyi, Guizhou, China

**Keywords:** primary lymphedema, whole-exome sequencing, Prox1, surgery, case report

## Abstract

**Background:**

Primary lymphedema (PL) is a chronic condition characterized by abnormal swelling of tissues due to impaired lymphatic drainage, leading to increased deposition of adipose tissue and fibrosis. Although several pathogenic variants in genes associated with PL have been identified, a significant number of cases remain unexplained, suggesting the possibility of undiscovered genetic links.

**Case presentation:**

This report describes a novel heterozygous mutation in the PROX1 gene (c.1019C>G, p.S340C) identified in a 59-year-old male patient with PL affecting both upper and lower extremities, indicating its potential role in lymphatic dysfunction. A comprehensive treatment strategy—combining conservative decongestive therapy for the less severely affected upper limb with radical reduction while preserving perforators (RRPP) and vascularized lymph node transfer (VLNT) for the severely affected lower limb—resulted in significant improvements in limb circumference, lymphatic transport, and overall quality of life.

**Conclusion:**

This report highlights the efficacy of combining RRPP and SC-VLNT in treating advanced-stage PL and emphasizes the importance of considering genetic factors in the management of this complex disease.

## 1 Introduction

Lymphedema is a progressive and debilitating condition resulting from compromised lymphatic fluid drainage, leading to the accumulation of lymph in the interstitial space. This accumulation triggers abnormal proliferation of adipose tissue and fibrosis, resulting in chronic localized swelling in the affected areas of the body. Primary lymphedema (PL) is relatively rare, affecting approximately 1 in every 10,000 individuals (Grada and Phillips, 2017), and arises from existing malformations or dysfunctions within the lymphatic system. The prevailing theory suggests a genetic basis for PL, with over 20 mutations linked to causative genes identified to date, alongside more than 10 candidate genes (Brouillard et al., 2021). However, only about 30% of PL cases can be associated with recognized pathogenic genes, underscoring the need for ongoing research to discover new genetic connections that could enhance diagnostic precision (Sung et al., 2022; Bonetti et al., 2022).

Prospero homeobox 1 gene (PROX1) serves as a crucial indicator of lymphatic endothelial cells (LECs) and is co-expressed with vascular endothelial growth factor receptor-3 (VEGFR-3) (Alitalo et al., 2005). It plays a significant role in the development and ongoing maintenance of the human lymphatic system throughout an individual’s life (Martin-Almedina et al., 2021). In 2020, rare variants of the PROX1 gene were identified in individuals suffering from PL (Ricci et al., 2020). It is hypothesized that these mutations may impair LECs functionality, thereby increasing the likelihood of disease manifestation. However, the pathogenicity of these variants has yet to be thoroughly confirmed, and PROX1 is still considered a candidate gene.

Recognizing the genetic modifications and associated syndromes that contribute to PL is essential for developing effective personalized treatments. Although significant advancements have been made in elucidating the genetic components associated with PL, current therapeutic methods are largely derived from those designed for secondary lymphedema (Ciudad et al., 2024). The primary strategy for treating PL emphasizes conservative approaches, particularly comprehensive decongestive therapy (CDT), which is widely regarded as the standard treatment due to its proven efficacy in reducing limb volume and preventing complications (Senger et al., 2023). However, more advanced stages of PL, such as elephantiasis, often necessitate surgical interventions (Wei et al., 2024). This case presents an unusual instance of primary lymphedema affecting both the upper and lower limbs, associated with a novel mutation in PROX1. Surgical interventions, including vascularized lymph node transfer (VLNT) and radical reduction while preserving perforators (RRPP), were performed on severe lower limbs, providing valuable insights into the diagnosis and treatment of this atypical condition.

## 2 Patient and methods

### 2.1 Patient

**FIGURE 1:**
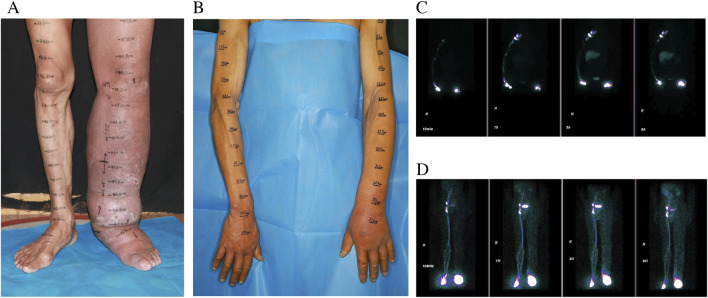
Preoperative pictures of LLL **(A)** LUL **(B)**, and preoperative lymphoscintigraphy images of LLL [**(C)** T6] and LUL [**(D)** T6].

A 59-year-old male was admitted to our hospital with septic shock. Prior to this admission, he had been evaluated at another facility, where limb amputation was recommended due to the severity of his condition. Initial treatment included a 2-week course of intravenous antibiotics and fluid resuscitation to stabilize the septic shock. Despite partial clinical improvement, persistent limb swelling and signs of severe infection prompted further investigation. Lymphoscintigraphy confirmed the presence of lymphedema, identified as the primary factor underlying the recurrent infection and subsequent septic shock (Figure 1).

### 2.2 Whole genome re-sequencing

Germline DNA was obtained from the peripheral blood samples of the patient and his relatives (Neither the patient’s father nor his son had PL or lymphatic disease.). Library preparation utilized the TruSeq Nano DNA LT Sample Preparation Kit (Illumina Inc., San Diego, CA, United States), and sequencing took place on the Illumina HiSeq X Ten platform. To detect pathogenic variants, a curated selection of genes associated with lymphedema was examined, which included the following: CCBE1, FOXC2, FLT4, KIF11, GATA2, SOX18, FAT4, PTPN14, GJC2, VEGFC, PIEZO1, PTPN4, CELSR1, HGF, MET, EPHB4, RASA1, ITGA9, PIK3CA, HRAS, SOS1, RAF1, RIT1, ADAMTS3, ANGPT2, ACVRL1, AKT1, ARAF, ARHGAP31, BRAF, CBL, CCM2, CTNNB1, DCHS1, ELMO2, ENG, FGFR1, GDF2, GLMN, GNA11, GNA14, GNAQ, IDH1, IDH2, KRAS, KRIT1, MAP2K1, MAP2K2, MAP3K1, MAP3K3, MAPK1, MAPK14, MAPK3, MTOR, NRAS, PDCD10, PDGFRB, PTEN, PTPN11, SHOC2, SMAD4, STAMBP, TEK, TP53, GJA1, and PROX1. The identified variants were cross-referenced with the dbSNP database (www.ncbi.nlm. nih.gov/SNP/) as well as the Human Gene Mutation Database (HGMD; http://www.biobase-international.com/product/hgmd). The pathogenicity of these variants was assessed using the Variant Effect Predictor (VEP) (http://www.ensembl.org/Tools/VEP) and MutationTaster tools. Additionally, minor allele frequencies (MAFs) were evaluated using the Genome Aggregation Database (GnomAD) (http://GnomAD.broad.institute.org/). The classification of variants was conducted following the guidelines set forth by the American College of Medical Genetics and Genomics (ACMG) (Richards et al., 2015).

### 2.3 Treatment

**FIGURE 2:**
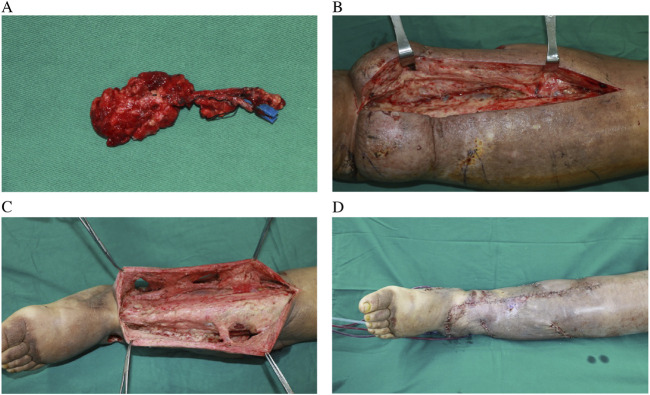
Harvested supraclavicular lymph node flap without skin paddle **(A)**, intraoperative view of severe fibrosis and skin thickening of LLL **(B)**, preserved perforator and tissue around them **(C)**, postoperative view of combination of RRPP and SC-VLNT **(D)**.

The treatment strategy for the patient was tailored according to the severity and progression of lymphedema in each limb. The milder upper limb lymphedema was managed conservatively through CDT. In contrast, the lower limb had developed severe fibrosis, necessitating a combined surgical approach that included RRPP and VLNT (Figure 2). This surgical procedure was performed under general anesthesia with a dual-team setup. One surgical team executed the RRPP, excising subcutaneous lymphedematous tissue along with the non-healing wound down to the deep fascia while meticulously preserving the perforators identified using a handheld Doppler device. Concurrently, the second team harvested a vascularized lymph node flap from the right supraclavicular area and subsequently transferred it to the left medial sural region using established techniques. Three days post-operation, we initially applied ordinary bandages for compression over a period of 3 days. Subsequently, we utilized gradient compression for a minimum of 6 months. Following this, we transitioned to the use of elastic socks.

### 2.4 Outcome evaluation

To objectively evaluate postoperative outcomes, limb volume was measured at baseline and during follow-up using the truncated cone formula, based on circumferential measurements. Infection frequency (episodes/year) was extracted from medical records, and quality of life was assessed pre- and post-operatively using the LYMQOL questionnaire for lower limb lymphedema.

## 3 Results

The individual exhibited gradual swelling of the entire left lower limb (LLL) and the left upper limb (LUL). The onset of swelling in the LLL occurred at the age of 7, while the swelling in the LUL emerged at the age of 49 (Figures 1A,D). Clinical evaluation categorized the LLL as Stage III and the LUL as Stage I, in accordance with the guidelines of the International Society of Lymphology (ISL) staging system. Lymphoscintigraphy revealed underdeveloped lymphatic structures in both affected limbs, characterized by the absence of lymph nodes and detectable lymphatic pathways. These observations were classified as total obstruction (T6) according to the Cheng Lymphedema Grading system (Figures 1B,C) (Cheng et al., 2018). The patient did not report any comorbid conditions, previous surgeries, or family history related to lymphedema or other congenital anomalies. However, there was a noted history of recurrent cellulitis, chronic ulceration, and lymphorrhea involving the LLL. Aside from this, the patient’s medical and social history appeared to be unremarkable.

Whole-exome sequencing, conducted on the patient, the patient’s father, and the patient’s son, identified a heterozygous mutation in the PROX1 gene present only in the patient. This mutation involved a nucleotide substitution at position 1019, changing cytosine (C) to guanosine (G) (c.1019C>G), resulting in a missense protein change (p.S340C) that is distinct from previously reported variants (Table 1). Computational pathogenicity analyses classified this mutation as “tolerated” by SIFT, “probably damaging” by PolyPhen, and “disease-causing” by MutationTaster.

**TABLE 1:** A list of PROX1 variants reported in studies.

Gender	Mutation	Mutation taster	PolyPhen	SIFT	Reported by
Male	c.1769T>A; p. (Leu590His)	Disease-causing	Probably damaging	Deleterious	Ricci et al. (2020)
Female	c.317G>A; p. (Gly106Asp)	Disease-causing	Probably damaging	Tolerated	
Male	c.1019C>G; p. (Ser340Cys)	Disease-causing	Probably damaging	Tolerated	This study

**FIGURE 3:**
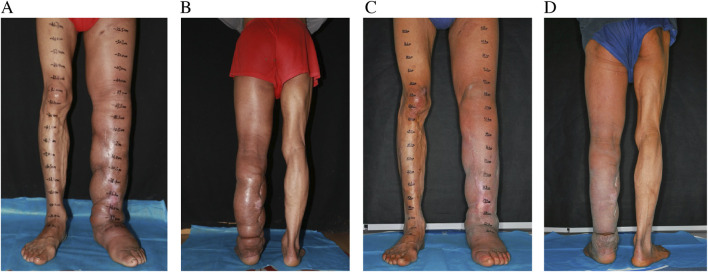
Postoperative pictures at 6 months **(A,B)** and 2 years **(C,D)** follow up.

The recovery process following surgery proceeded smoothly, demonstrating prompt reductions in limb size. The patient was discharged from the hospital on the 25th day post-surgery, with instructions to maintain compression therapy for a minimum of 6 months. Follow-up evaluations at 6 and 24 months showed a reduction in LLL limb volume by 1354 mL and 2482 mL, respectively. The patient experienced no recurrence of cellulitis during the 2-year follow-up period, compared with three documented episodes per year prior to surgery. The LYMQOL questionnaire demonstrated improvements across all domains: function (from 21 to 10), appearance (from 7 to 3), symptoms (from 11 to 5), and mood (from 8 to 4). Clinically, the patient reported enhanced mobility, reduced limb heaviness, and improved confidence in appearance and daily functioning. No complications, including donor site lymphedema, were observed during follow-up assessments (Figure 3).

## 4 Discussion

Traditionally, PL has been categorized based on the age at which it manifests: congenital (before 2 years), praecox (ages 2–35), and tarda (over 35 years). This classification system, however, provides limited insight into the genetic factors or related syndromes involved. To address this shortcoming, contemporary classification systems are now incorporating genetic and phenotypic elements (Gordon et al., 2020), including conditions such as Parkes-Weber syndrome (associated with RASA1), lymphedema-distichiasis syndrome (FOXC2), Milroy disease (FLT4), and Emberger syndrome (GATA2). Nonetheless, many types of lymphedema lack a well-defined genetic basis, underscoring the need for further investigations to identify new gene associations (Sudduth and Greene, 2022). Among the various genetic possibilities, mutations in the PROX1 gene are particularly significant due to their essential role in the development and maintenance of the lymphatic system. PROX1 functions as a transcription factor that is crucial for several organ systems and plays a key role in the lymphatic system by promoting the expression of LEC-specific markers and preserving the identity of LECs. Studies involving animal models have shown that the absence of PROX1 results in the failure to form a lymphatic network, leading to embryonic death by day 15 (Wigle et al., 2002). Mice with one functional copy of PROX1 can develop lymphatic networks but exhibit neonatal mortality, indicating that two functional PROX1 copies are essential for survival (Wigle and Oliver, 1999). Notably, individuals with heterozygous PROX1 mutations can often live to around 60 years, suggesting the existence of compensatory mechanisms and highlighting the importance of further elucidating the gene’s implications in PL.

Although PROX1 has long been recognized for its role in lymphatic development, few human studies have directly linked PROX1 mutations to primary lymphedema. Most genetic research in PL to date has focused on genes like FLT4, FOXC2, and GJC2, while PROX1 remains underrepresented in clinical cohorts. The identification of a novel, heterozygous PROX1 mutation in this patient adds to a limited but emerging body of evidence implicating PROX1 in both syndromic and non-syndromic forms of lymphedema. This case provides important real-world validation for hypotheses developed in animal models and supports the concept of genetic heterogeneity in PL, where atypical or late-onset phenotypes may involve underrecognized gene pathways.

In this instance, the individual exhibited lymphedema in the LLL at the age of 7, which subsequently progressed to the LUL at age 49—a notably rare and unusual progression. A literature review uncovered only a single analogous case of unilateral lymphedema affecting both upper and lower limbs (Liu et al., 2021). Whole-exome sequencing identified a heterozygous variant in the PROX1 gene (c.1019C>G, p.S340C), which was deemed “probably damaging” by PolyPhen and classified as “disease-causing” by MutationTaster. It is proposed that this variant may impair lymphatic drainage, rendering the system more susceptible to decompensation in response to factors such as inflammation or injury. The development of PL in this individual exemplifies the interaction between inside genetic predispositions and outside environmental influences (Yoshida et al., 2021). The initial involvement of the lower limb may be exacerbated by gravitational factors that worsen lymphatic insufficiency (Razavi et al., 2024), while the subsequent onset of lymphedema in the upper limb later in life may indicate a decline in lymphatic function associated with aging. Research using animal models has demonstrated that aging can lead to decreased lymphatic pump efficiency (Karaman et al., 2015), reduced frequency of vessel contractions, and lower density of lymphatic vessels, reinforcing the concept of a multifactorial basis for PL. The relationship between the age of onset and the severity of the disease merits further exploration. Earlier occurrences of PL frequently correlate with more severe progression and increased morbidity (Senger et al., 2023), as evidenced in this case, where the involvement of the LUL at a later age was significantly less severe than the earlier and more intense presentation of LLL. This contrast underscores the accumulating impact of lymphatic dysfunction over time in cases with an earlier onset, highlighting the necessity for tailored strategies that consider the chronology of the disease.

Although the surgical plan was primarily based on clinical staging and lymphoscintigraphy, the patient’s atypical disease course—early-onset lower limb lymphedema followed by late-onset upper limb involvement—and the near-complete absence of functional lymphatics on imaging strongly suggested a systemic, likely genetic etiology. Literature review indicated that combined upper and lower limb involvement in primary lymphedema is extremely rare, further supporting a congenital origin (Liu et al., 2021; Taywade and Sethi, 2020; Mrhac et al., 2018). To explore this, preoperative whole-exome sequencing was performed, although results became available only after surgery. A heterozygous PROX1 variant, predicted to be pathogenic by multiple *in silico* tools, was identified. While unreported in humans, this variant aligns with animal studies showing PROX1 is essential for lymphatic endothelial identity and vessel formation; gene knockout models exhibit complete lymphatic failure and embryonic lethality. This genetic finding retrospectively supported our decision to avoid procedures like lymphaticovenous anastomosis (LVA), which rely on preserved lymphatic pathways. Instead, we adopted a combined strategy of VLNT and RRPP, targeting both functional restoration and removal of fibrofatty tissue. Although genetic information alone does not yet dictate the choice of surgical approach, in this case it provided valuable *post hoc* confirmation of a system-wide dysfunction and highlighted the potential of incorporating genetic screening in future individualized treatment planning for primary lymphedema.

Notably, while systemic lymphatic dysfunction might theoretically reduce the effectiveness of VLNT, recent studies have shown that VLNT can still be beneficial in such contexts. Maruccia et al. (2023) provided histological evidence of neolymphangiogenesis surrounding transferred lymph nodes. Similarly, long-term volume reduction and functional improvements have been reported even in advanced-stage or genetically associated primary lymphedema cases (Sakarya et al., 2022; Yodrabum and Poungjantaradej, 2023). Losco et al. (2022) further demonstrated that combining VLNT with excisional procedures offers synergistic benefits by both restoring lymphatic function and resolving fibrofatty tissue burden. These findings support the rationale behind our choice of VLNT combined with RRPP, even in the setting of a PROX1 mutation.

Previous research has demonstrated that various combined surgical strategies for PL, including RRPP + VLNT, liposuction (LS)+ lymphaticovenous anastomosis (LVA), and LS + VLNT, yield encouraging results in terms of limb volume reduction and functional enhancement (Gaxiola-García et al., 2024). However, a clear consensus on the management of PL remains elusive. The LUL, diagnosed with Stage I PL, was treated with CDT to reroute lymphatic fluid, in conjunction with compression therapy to prevent re-accumulation (Onoda and Nishimon, 2021). Over 2 years of CDT, this approach resulted in a substantial reduction in limb volume, improved skin condition, and enhanced quality of life. For the LLL exhibiting advanced-stage PL, surgical options become critical. While LVA is a recognized physiological choice, it requires the presence of functional lymphatic vessels. In this case, indocyanine green lymphography indicated an absence of functional LVs, making VLNT the preferred physiological intervention. This technique enhances the uptake of lymphatic fluid via natural lymphovenous connections within the transplanted flap, or it encourages lymphangiogenesis by newly transplanted healthy lymphatic tissue. Although VLNT may restore lymphatic function, it does not address the extensive fibrotic and fatty tissue associated with advanced lymphedema. In contrast, RRPP synergizes with VLNT by effectively removing fibrotic tissue while preserving perforators to ensure adequate vascular supply and minimize the risk of complications.

This case exemplifies the effectiveness of combining RRPP with VLNT in advanced-stage PL. Accurate diagnosis through both genetic assessments and clinical evaluations is essential for tailoring treatment strategies. Although mutations in PROX1 represent a potential genetic target, further studies involving larger patient cohorts and comprehensive functional analyses are required to validate their role in the pathogenesis and physiology of PL, thereby aiding in the development of targeted precision treatments.

## Data Availability

The raw data supporting the conclusions of this article will be made available by the authors, without undue reservation.
